# A Dictionary of the Terms Used in Medicine and the Collateral Sciences

**Published:** 1845-01

**Authors:** 


Art. VIII.—*A Dictionary of Terms used in Medicine and the Collateral Sciences.* By R. D. Hoblyn, a.m. Oxon. Second Edition, revised and enlarged.—*London*, 1844. 8vo, pp. 394.

We gave a very favorable account of this little book on its first appearance, (Br. and For. Med. Rev. vol. I, p. 517,) and we have only to repeat the same, with increased emphasis. It is, for its size, decidedly the best book of the kind, and ought to be in the possession of every student. Its plan is sufficiently comprehensive, and it contains an immense mass of necessary information in very small compass.

